# A Literature Review on SARS-CoV-2 and Other Viruses in Thyroid Disorders: Environmental Triggers or No-Guilty Bystanders?

**DOI:** 10.3390/ijerph20032389

**Published:** 2023-01-29

**Authors:** Francesca Gorini, Cristina Vassalle

**Affiliations:** 1 Institute of Clinical Physiology, National Research Council, 56124 Pisa, Italy; 2 Fondazione Gabriele Monasterio CNR-Regione Toscana, 56124 Pisa, Italy

**Keywords:** COVID-19, SARS-CoV-2, vaccine, virus, thyroid, thyroid disorder, thyroid dysfunction subacute thyroiditis, nonthyroidal illness syndrome, autoimmune thyroid diseases, Graves’ disease, Hashimoto disease

## Abstract

A growing number of findings indicate a relationship between COVID-19 infection and thyroid dysfunction. This association is also strengthened by knowledge on the potential of viral infections to trigger thyroid disorders, although the exact underlying pathogenetic process remains to be elucidated. This review aimed to describe the available data regarding the possible role of infectious agents, and in particular of SARS-CoV-2, in the development of thyroid disorders, summarizing the proposed mechanisms and levels of evidence (epidemiological, serological or direct presence of the viruses in the thyroid gland) by which the infection could be responsible for thyroid abnormalities/diseases. Novel data on the association and mechanisms involved between SARS-CoV-2 vaccines and thyroid diseases are also discussed. While demonstrating a clear causal link is challenging, numerous clues at molecular and cellular levels and the large amount of epidemiological data suggest the existence of this relationship. Further studies should be taken to further investigate the true nature and strength of this association, to help in planning future preventive and therapeutic strategies for more personal and targeted care with attention to the underlying causes of thyroid dysfunction.

## 1. Introduction

A properly functioning thyroid gland is crucial for health, influencing growth, neuronal development, reproduction and as a key regulator of energy metabolism, although thyroid disorders/diseases are extremely common and affect 200 million people worldwide [1].

Viral infection may represent one of the major environmental factors related to common thyroid disorders, including subacute thyroiditis (SAT), nonthyroidal illness syndrome (NTIS) and autoimmune thyroid diseases (AITDs) including Graves’ disease (GD) and Hashimoto’s thyroiditis (HT) (Table 1).

In this context, a number of findings have indicated a relationship between a variety of viruses and thyroid abnormalities/diseases, even though the available published evidence is still inconclusive and too sparse to definitively prove a viral etiology [6,7]. In fact, it is not yet clear whether viruses act as bystanders or whether, through molecular mimicry, they activate the inflammatory cellular immune response and as such elicit thyroid autoimmunity, with adverse events persisting even after the infection has cleared.

The coronavirus disease 19 (COVID-19) pandemic has introduced SARS-CoV-2 as a novel additive and potent environmental trigger possibly implicated in thyroid dysfunction. Globally, as of 19 January 2023, there have been 663,248,631 confirmed cases of COVID-19, including 6,709,387 deaths, reported to the World Health Organization [8]. As of 17 January 2023, a total of 13,131,550,798 vaccine doses have been administered [8]. Actually, as the pandemic was spreading, emerging evidence was reported on the direct consequences of SARS-CoV-2 on various thyroid disorders such as SAT, NTIS and AITDs [6,7]. The amount of information now available may shed light on the matter and could help answer questions that remain unresolved in the relationship between viruses and thyroid pathophysiology.

In fact, a number of narrative and systematic reviews have been published with the aim of summarizing the epidemiology and major clinical symptoms of thyroid dysfunction events during SARS-CoV-2 infection and attempting to indicate their clinical management [9,10,11,12]. However, according to a systematic review of seven studies and 1237 patients with COVID-19, the prevalence of thyroid dysfunction, defined as abnormalities in levels of the thyroid stimulating hormone (TSH) and/or thyroid hormones, is highly variable in COVID-19 disease, ranging from 13 to 64% [13]. Nonetheless, a growing body of evidence has recognized that, although the leading cause of death associated with COVID-19 is acute respiratory distress syndrome, the pathogenesis of COVID-19 is not limited to the upper respiratory tract and lungs, and may affect multiple organ systems, including the thyroid gland [10,14,15].

**Figure 1:**
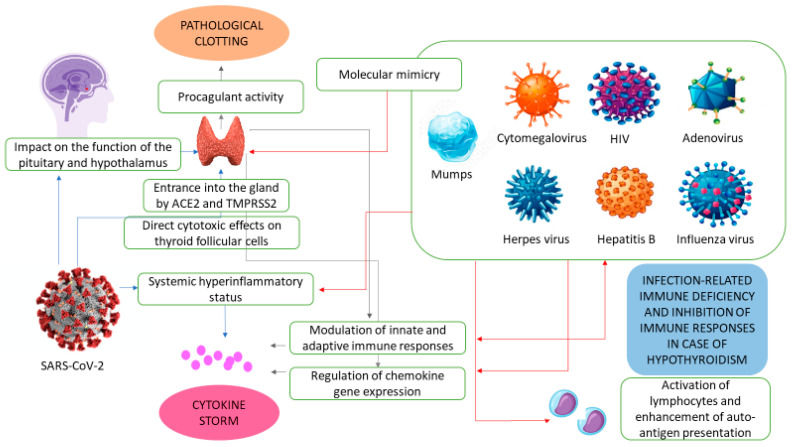
Main proposed mechanisms underlying the relationship between SARS-CoV2-2 and other viruses and the thyroid gland. Blue arrows indicate actions performed by SARS-CoV2-2; gray arrows indicate actions performed by the thyroid; red arrows indicate actions performed by other viruses. See text for details. Abbreviations: ACE2: angiotensin-converting enzyme 2; TMPRSS2: transmembrane serine protease 2.

Angiotensin-converting enzyme 2 (ACE2), the receptor able to bind spike proteins of SARS-CoV-2, allowing the virus to enter host cells, is not only expressed on the surface of pneumocytes, but also at high levels in the thyroid [16,17]. The thyroid further produces transmembrane serine protease 2, a protein critical to enable SARS-CoV-2 cell access [18]. Therefore, researchers have speculated that the virus may affect the gland by direct cytotoxic effects on thyroid follicular cells or, alternatively, through an abnormal immune regulation, the so-called “cytokine storm”, which consists of a systemic hyperinflammatory status, resulting in thyroid dysfunction [12,19,20,21]. Notably, the thyroid hormones L-thyroxine (T4) and 3,3′,5-triiodo-L-thyronine (T3) are modulators of immune responses (both innate and adaptive) through both genomic and nongenomic mechanisms [22]. In addition, according to a further pathophysiological mechanism hypothesized to facilitate SARS-CoV-2 internalization and involving the binding of thyroid hormones to plasma membrane integrin αvβ3, T3 and T4 may regulate chemokine gene expression and thus contribute to trigger inflammatory processes that are a hallmark of systemic viral infections [6,23]. Finally, thyroid hormones are involved in procoagulant activities and stimulate platelet-endothelial cell interaction that helps lead to the pathological clotting which occurs during COVID-19 infection [23,24]. Of note, it should be remembered that the thyroid gland does not govern itself, but acts through the entire hypothalamic–pituitary–thyroid (HPT) axis and that SARS-CoV-2 has an impact on the function of the pituitary and hypothalamus [25]. Indeed, not only SARS-CoV-2 has a substantial effect on TSH-secreting cells, resulting in lower TSH concentrations and, consequently, a disruption of pituitary endocrine axis feedback loops, but the suppression of the HPT axis could be a common complication in COVID-19 patients and an indicator of the severity of prognosis [25,26] (Figure 1).

This review aims to discuss the available evidence related to the possible role of infectious agents in the development of thyroid disorders (with particular attention to SARS-CoV-2 infection, the diffusion of which has provided an increasing amount of data on this relationship as well as on SARS-CoV-2 vaccination), summarizing the mechanisms and levels of evidence (epidemiological, serological or direct presence of the viruses in the thyroid organ) by which the infection could trigger thyroid abnormalities/diseases. This knowledge would be useful for planning future preventive and therapeutic strategies for more personal and targeted care with attention to the underlying causes of thyroid dysfunction.

## 2. Virus Infection and Thyroid Function/Dysfunction

SAT is an inflammatory disorder of the thyroid often causing thyrotoxicosis, more prevalent in the female sex. Clinical presentation contains symptoms including neck pain and thyroid tenderness, and different typical characteristics of a viral infection, following a triphasic presentation: first thyrotoxicosis (i.e., high free T4 levels and low to undetectable TSH levels), then hypothyroidism, which is transient in 90–95% of cases, and lastly normal thyroid function (with the exception of 5–10% of cases with permanent hypothyroidism and requiring thyroid replacement therapy with levothyroxine—LT4) [11,27,28]. The incidence of SAT has been shown to have a seasonal pattern in parallel with outbreaks of viral infection (e.g., enterovirus, influenza), providing an early clue to the interaction between viral infection and thyroid function [29,30]. Although SAT is a thyroid inflammatory disease whose pathogenesis has not yet been fully elucidated, viral infections have been established as among a major cause in genetically predisposed individuals [25]. In fact, many types of viruses have been related to SAT, including Coxsackie viruses, adenoviruses, mumps, HIV, and Epstein-Barr virus [29,31,32]. On the other hand, SAT may also represent a very rare and as such largely under-recognized complication after dengue virus infection [33].

Indeed, in addition to the several proposed mechanisms for thyroid organ damage including secretion of pro-inflammatory cytokines and chemokines, infection-related immune deficiency, lymphocyte destruction, inhibition of innate immune response, and direct destruction of follicular cells with apoptosis, SAT susceptibility is closely associated with the expression of some human leukocyte antigen (HLA) haplotypes [34,35,36] (Figure 1). In particular, HLA-B*18:01 and HLA-DRB1*01 represent independent SAT risk alleles, while HLA-B*35 and HLA-C*04:01 alleles have been shown to be markers of genetic susceptibility to SAT independently, whether present alone or together [36].

SAT may also occur after vaccination (as in the case of influenza), or be related to antiviral drugs as in the case of SAT related to chronic hepatitis C virus (HCV) infection, which has been linked to effects of interferon alpha treatment [29,37,38,39].

Another alteration of the thyroid is NTIS, also known as euthyroid sick syndrome or low T3 syndrome, which occurs in the absence of overt thyroid disease but in a context of serious illnesses, and is characterized by an initial reduction in plasma T3 and by increased plasma reverse T3 levels without a concomitant rise in TSH [40]. Conditions of persistent illnesses, despite low T3 and T4, lead to a global reduction of TSH level, free T3 (fT3) and free T4 [41,42]. NTIS is present in most critically ill patients, being considered a strong predictor of poor prognosis and death in several systemic conditions (e.g., major trauma, surgery, respiratory failure, septic shock, cerebrovascular and cardiovascular diseases, cancers, lymphoproliferative diseases) [42,43]. Nonetheless, although the extent of NTIS is associated with prognosis, whether hormonal changes reflect a protective mechanism or a maladaptive process during prolonged illness remains to be established [42]. For instance, in HIV infection, NTIS is a common finding, and its presence correlates with a worse prognosis [44,45,46]. Moreover, low T3 syndrome may induce a worse prognosis in patients with hepatitis B virus-related acute-on-chronic liver failure [47].

Thyroid function abnormalities can also be observed in the antiretroviral therapy course, which make the thyroid screening in HIV-infected patients a reasonable strategy [44,48].

Since thyroid hormones are key regulators of innate and adaptive immune responses, the condition of hypothyroidism increases the risk of contracting viral infections which, in turn, are cited as environmental factors involved in subacute and autoimmune thyroiditis, although not directly with hypothyroidism [29,49]. In general, infectious agents, in particular viruses, are believed to be capable of triggering or worsening autoimmunity in genetically predisposed individuals [50,51]. AITDs, the result of a complex interaction between genetic and environmental determinants, define conditions in which the thyroid gland is progressively damaged through an autoimmune destructive process [52,53,54]. Many risk factors have yet to be defined, although several viruses have been associated with AITDs. GD is an immune system disorder characterized by enlargement of the thyroid gland (goiter) and overproduction of thyroid hormones (hyperthyroidism), while HT is an autoimmune thyroid disease for which the exact etiology remains unclear with, as a typical sign, the infiltration of autoreactive T cells in thyroid tissues and the production of autoantibodies (anti-thyroid peroxidase—TPO and anti-thyroglobulin—TG) associated with destruction of the thyroid follicles, which ultimately results in hypothyroidism (i.e., low serum free T4 and elevated serum TSH levels) [12,55,56]. The pathogenesis of HT therefore differs from GD both in clinical manifestations (hypothyroidism vs. hyperthyroidism) and in the main type of autoimmunity involved (cell-mediated vs. humoral) [57,58]. In particular, infections from viruses such as human herpes virus, cytomegalovirus, Epstein-Barr and HCV as well as interferon treatment, have been identified as triggers of HT, by activating innate and adaptive immunity [59,60,61,62,63,64]. Additionally, HCV, influenza virus and Epstein-Barr virus have been associated with GD, while HCV and Helicobacter Pylori (H. pylori, a microorganism although not a virus) may be related to both HT and GD [65,66,67,68,69].

Nonetheless, the available findings are still sparse, the data at different levels of evidence (epidemiological, serological or direct evidence of virus presence in the tissue) sometimes conflicting, therefore still far from confirming a close cause–effect relationship without any doubt, which is however difficult to be definitively proven [29,62]. Certainly, neither the relationship with clinical status (e.g., same prevalence of the viral pathogens among AITDs patients and controls) and the role of coinfection, nor the presence of susceptibility genes, which may increase the risk of thyroid dysfunction, are easily demonstrable [70]. Specifically, most studies rely on serological measurements, whereas the presence and copy numbers of viral genomes may best reflect real-time infection activity. Indeed, antibodies do not directly reflect viral status, and elevated levels may persist long after viral eradication, without a close correlation with disease severity or duration, thus this information is difficult to interpret. The presence of antibodies against a virus does not mean that this infection necessarily causes disease, especially if the virus is common in the population. Conversely, a virus can be cleared from the infected organisms without leaving any signs other than the presence of specific antibodies. Furthermore, the infectious agent can remain in the tissue without any evidence of systemic presence. The interaction between genetic asset and infection is remarkable, as in genetically susceptible patients some genes are expressed after infection, explaining why common viruses may elicit autoimmunity only in some (susceptible) subjects but not in others.

It is otherwise true that infectious agents may be held up as culprits of autoimmune diseases through several possible mechanisms, for which the equilibrium between the two counterparts represented by the host (e.g., susceptibility genes, immune responses) and the virus (e.g., viral properties, viral strain, viral load) is crucial. Table 2 summarizes the pros and cons in the relationship between infection from viruses and thyroid disorders. The main mechanisms proposed to explain this relationship include molecular mimicry (the virus may mimic the structure of some components of the thyroid, triggering autoimmune responses), enhanced processing and the presentation of auto-antigens by antigen-presenting cells (e.g., changes in self antigen expression), increased inflammation and cytokine release (activation of autoreactive T cells) and lymphocyte activation by lymphotropic viruses (e.g., increased generation of circulating immune complexes) (Figure 1, Table 2) [62]. For example, the main pathogenetic events related to H. pylori in association with GD and HT, have been identified in enhanced inflammatory processes and molecular mimicry [71]. Besides, different common viruses have been identified in the thyroid tissue (e.g., enterovirus, parvovirus, herpesvirus) [70].

## 3. SARS-CoV-2 and Thyroid Function/Dysfunction

### 3.1. Subacute Thyroiditis

From the first clinical case of SAT (also known as De Quervain thyroiditis or granulomatous thyroiditis) associated with COVID-19 published in May 2020 [27], a large number of SAT cases have been progressively reported [9,11,15]. The incidence of non-coronavirus SAT has been estimated at 12.1 cases/100,000 per year, with a female/male ratio of 5:1 [11]. Based on a systematic review of 15 studies (case reports and case series) published between December 2019 and February 2021, on 17 subjects, Christensen and colleagues (2022) [28] estimated that patient ages generally ranged from 18 to 69 years in COVID-19-associated SAT. Comparable results were found in a narrative review by Popescu et al. (2022) [11] including 2 retrospective studies, 5 case series, and 29 case reports (a total of 81 patients), which reported that the youngest patient was 18 years old and the oldest 85 years old, albeit using a postmortem diagnosis [72]. The majority of cases with typical thyroiditis were females [11,29], however the precise incidence of SAT among subjects infected with COVID-19 virus is currently indefinite [10]. Consistent with the Classical-SAT that typically follows a viral infection, the time recorded from diagnosis of COVID-19 infection and symptoms of SAT was usually from six to eight weeks (and up to six months), though SAT also occurred simultaneously with COVID-19 disease, and even in the absence of respiratory symptoms [11]. Indeed, in a systematic review comprising 38 patients from 26 case reports, case series, and letters on SAT associated with COVID-19 and published by the end of 2021, Ando et al. (2022) showed that SAT may develop regardless of the severity of COVID-19, and in most patients with SAT, infection from SARS-CoV-2 was classified as mild or moderate in severity [10]. Similar findings were reported in the analysis by Popescu et al. (2022), with SAT associated with various COVID-19 presentations, from asymptomatic to complicated forms [11].

In the majority of cases, the clinical picture appeared similar to that reported for typical SAT cases, with fever, anterior neck pain, fatigue, tremors, anosmia, sweating, and palpitations characterizing thyrotoxicosis, which results from destruction of thyroid follicles and release of thyroid hormones [9,73]. In addition to clinical symptoms, specific laboratory and imaging features of SAT included thyroid high erythrocyte sedimentation rate (ESR), common elevation of C-reactive protein (CRP), increase in white blood count, negativity for thyroid autoantibodies, enlarged hypoechoic thyroid with decreased vascularity found on ultrasound, and markedly reduced or absent iodine uptake in the gland as detected by thyroid scintigraphy [9,28]. In the systematic review by Stasiak and Lewiński (2021) [9] including 73 studies, 5 of them reported cases of painless SAT, therefore neck pain, which is considered as the key diagnostic criterion, may also be absent in the COVID-19-associated disorder, probably as a consequence of the frequent use of analgesics and nonsteroidal anti-inflammatory drugs (NSAIDs) in COVID-19 patients [74,75]. In particular, in one of the first published case series, Muller et al. (2020) [75] documented that 75% of the eight patients with thyroiditis/thyrotoxicosis after COVID-19 infection requiring high intensity of care and followed up at a mean of 55 days, had ever experienced neck pain, and instead of lymphocytosis, displayed the lymphopenia occurring in COVID-19 patients. Furthermore, these patients had low or suppressed levels of TSH with normal levels of T3 and T4, indicating that SAT may have overlapped with NTIS, a condition described as thyroxine thyrotoxicosis [75]. Lymphopenia in COVID-19 may be triggered by various processes including a direct effect of the virus on the apoptosis of lymphocytes and bone marrow impairment, cytokine-induced lymphocyte apoptosis, metabolic and biochemical abnormalities resulting in decreased production, functionality and survival of lymphocytes, all postulated mechanisms that in turn can influence the HPT axis [76]. Thus, lymphopenia, a marker of extrapulmonary signs in COVID-19, not only can predict the onset of thyroid dysfunction but the lack of lymphocytic infiltration and the formation of giant cells (congregates of lymphocytes, histiocytes, and colloid) in the thyroid prevents tension in the thyroid capsule and appearance of pain in early post-COVID SAT [75,77]. In SAT that occurs later, after normal lymphocyte counts have recovered, the resulting lymphocyte infiltration can rapidly lead to pain in the anterior cervical region [77].

Consistently, in a recent combined retrospective–prospective study conducted over a period of 20 months, among 6.8% of patients with COVID-19 developing SAT, those with painless SAT (n = 5) presented earlier, had more severe thyrotoxic manifestations and exhibited higher CRP, interleukin-6 (IL-6) and neutrophil-lymphocyte ratio (NLR) and lower absolute lymphocyte count than those with painful SAT (n = 6) [78]. Besides, the authors reported significant correlations of IL-6 with free and total T4 and free and total T3, suggesting that destructive effects of cytokines may play a key role in painless SAT [78].

Similar results were observed in a retrospective single-center study, in which the close relationship between thyrotoxicosis and higher serum IL-6 in patients hospitalized for COVID-19 in non-intensive care units, is indicative of the cytokine storm action associated with COVID-19 in triggering and sustaining gland inflammation [74]. In this study, however, contrary to typical SAT parameters, 74.6% of patients with thyrotoxicosis (20.2% of the total) had normal TSH levels, while subjects with overt thyrotoxicosis exhibited higher serum free T4 levels than those with a subclinical disorder [74]. A cohort study instead reported that, in a small subset of COVID-19 survivors (n = 55) followed up with a median of 79 days, none had overt thyrotoxicosis (using a conservative definition of TSH < 0.30 mU/L and free T4 > 23.0 pmol/L), even patients admitted to the intensive care unit [79]. Overall, these data suggest that COVID-19 infection gives rise to a novel entity of SAT, which could often be underdiagnosed also due to its self-limiting nature and especially in subjects with severe COVID-19 disease, where multiple manifestations of infection may hide inflammation of the thyroid gland [11,80]. On the other hand, in most post-viral forms, SAT may present as a first or unique manifestation of SARS-CoV-2 infection or, alternatively, within a clinic picture dominated by SAT rather than COVID-19 disease [11]. Of note, thyrotoxicosis may increase the cardiovascular risk even after short exposure to thyroid hormone excess, favoring the development of arrhythmias and thromboembolic events described in patients with SARS-CoV-2 infection, aggravating the general status of severe infections [35,74,81,82]. After a diagnosis of COVID-19-induced SAT, patients should be treated with beta blockers and NSAIDs, while the efficacy of antithyroid drugs during the thyrotoxic stage of the disease has not been demonstrated [11,28]. Actually, most patients with SAT are treated with glucocorticoids, which are effective at relieving symptoms and reducing the incidence of relapse [25,83]. Glucocorticoids, now considered the gold standard in the treatment of COVID-19 patients requiring invasive mechanical ventilation or oxygen supplementation [84], may instead lead to a decrease of serum TSH through the direct release suppression of the TSH-releasing factor in the hypothalamus, providing both an additional mechanism of thyroid dysfunction and delaying the diagnosis of SAT [18,25,85].

### 3.2. Nonthyroidal Illness Syndrome

NTIS has been frequently observed in COVID-19 patients. Chen and colleagues [85] reported that 56% of patients with COVID-19 had lower TSH than the normal range and serum TSH levels in the severe and critical patient group were significantly lower compared to no-COVID-19 pneumonia patients with a similar degree of severity. In addition, the degree of decrease in TSH and total T3 levels was positively correlated with disease severity [85]. Consistently, TSH and fT3 were significantly lower in COVID-19 deceased patients than in those moderately to severely ill, or critically ill but recovered [86] as well as in living COVID-19 patients, compared to healthy controls [16]. A study by Gong et al. (2021) [87] reported that among 150 patients with COVID-19 and low fT3 levels, those in the low TSH group had higher mortality and critical illness rates compared to those in the normal TSH group, and low TSH levels were independently associated with 90-day mortality. The mortality rate was also significantly higher in the low fT4 group [86]. Although these findings might suggest peculiar effects of COVID-19 on TSH levels, both directly on the pituitary secreting cells and indirectly on the pituitary-thyroid axis as a consequence of systemic inflammation caused by virus infection, the observed decrease in TSH level could be induced by hypoxemia or by glucocorticoids with which most patients were treated [85], as previously described. In contrast, Muller et al. (2020) [75] found that patients admitted to high intensive care units(HICUs) because of COVID-19 had lower TSH levels than those admitted to HICUs in the absence of COVID-19 and those in the group with COVID-19 but admitted to low intensive care units, while no significant difference was found in fT3 levels. Importantly, low concentrations of TSH and T3 in the HICU group were associated with normal or elevated concentrations of T4, indicating a transient thyroxine increase related to the thyrotoxicosis, in the underlying context of NTIS [75]. NTIS was also experienced by patients with mild to moderate COVID-19 not requiring intensive care, with low fT3 having prognostic significance as associated with worsening clinical severity of COVID-19 [88]. The analysis of the clinical data of 100 patients with COVID-19 showed that severely or critically ill patients had lower concentrations of serum albumin, fT3, TSH and fT3/free FT4 than non-severely ill patients, and their fT3 reduction was independently associated with all-cause mortality [89]. Moreover, subjects with low fT3 had lower fT3/free T4 ratio, higher inflammatory markers (CRP and ESR) and higher indices of tissue injury (i.e., aspartate aminotransferase and lactate dehydrogenase—LDH), while the inverse correlation between ESR and fT3/free T4 ratio was indicative of the effect of systemic inflammation on the deiodinase activity [90]. Similar results were found in patients with COVID-19 and NTIS (27.5% of the total), who were characterized by higher levels of ESR, CRP and procalcitonin, and a lower lymphocyte count than in COVID-19 non-NTIS patients [91]. Conversely, in a study by Gao et al. (2020) [89], fT3 was negatively associated with CRP and IL-6 only in non-severely ill patients and survivors, and with tumor necrosis factor alpha (TNF-α) only in survivors, while not related to CRP, IL-6 and TNF-α in non-survivors, possibly due to a different role of the inflammatory response in NTIS at different stages of COVID-19. Recently, Lui and co-authors (2022) [76] reported that in COVID-19 patients with fT3 levels compatible with NTIS, TSH showed a significant correlation with lower lymphocyte count, suggesting a potential interaction between the HPT axis and the immune system, which is supported by the parallel recovery in TSH and fT3 with lymphocyte counts in those subjects reassessed after a median of nine days. In addition, although patients who had both NTIS and lymphopenia were more likely to have severe COVID-19 outcomes compared to those who only had either one of NTIS or lymphopenia, only NTIS was an independent predictor of severe outcomes in COVID-19 [76]. Sciacchitano et al. (2021) [92] showed that, among patients hospitalized for COVID-19, low fT3 levels (61.2% of the total) were significantly associated with increased NLR and absolute neutrophil count and with reduced levels of T lymphocytes, especially of the helper-inducer T cell subpopulations that were observed in the more severe group of patients. Low fT3 values were also correlated with increased levels of inflammation (high-sensitivity CRP), tissue damage (LDH, ferritin, high-sensitivity cardiac troponin I) and coagulation (prothrombin time, fibrinogen, D-dimer) serum markers, as well as with higher radiological scores of disease severity (Lung Immune Prognostic Index, Sequential Organ Failure Assessment Score and Tomographic severity score), clearly indicating that reduced fT3 levels can be considered as a prognostic biomarker of COVID-19 [92]. Of interest, the authors also noted a different expression pattern of a small subset of genes (i.e., CD38, CD79B, IFIT3 and NLRP3) involved in the immune reaction and expressed in immune cells in two patients that, in addition to COVID-19, also presented hematological malignancies [92]. In a subsequent study, Sciacchitano et al. (2021) [93] evaluated the thyroid hormone function and body composition by Bioelectrical Impedance Analysis in 74 critically ill COVID-19 patients and in 96 outpatients affected by thyroid diseases in different functional conditions. In patients with COVID-19, a significant inverse correlation was observed between fT3 serum levels and the hydration status (through the Total Body Water/Free Fat Mass—TBW/FFM ratio). Furthermore, reduced fT3 serum values in COVID-19 patients were associated with the increase in TBW, extracellular water and sodium/potassium exchangeable ratio (Nae:Ke), and with the reduction of the intracellular water. Conversely, these alterations were not seen in non-COVID-19 patients, except for the single patient affected by severe hyperthyroidism and myxedema. In particular, Na+-K+ pump could represent a possible target of T3 action, as patients with COVID-19 with NTIS had lower mRNA expression levels of the genes coding for the two major isoforms of this pump. Overall, the results indicate that low fT3 serum levels may lead to the altered distribution of salt and water in the body as a consequence of reduced peripheral thyroid hormone activity and a clinical picture resembling that observed in myxedema [93].

Importantly, more than 90% of children with COVID-19 disease in a picture of severe multisystem inflammatory syndrome exhibited NTIS; however, variables related to organ damage, inflammation and severity of multisystemic involvement and other hematological parameters, such as hemoglobin, platelets, leukocytes, were unrelated to thyroid function, possibly suggesting that low T3 syndrome may act as an adaptive mechanism to conserve energy during a long period of critical illness rather than during acute events [94].

### 3.3. Autoimmune Thyroiditis

A number of case reports, case series, and observational studies have investigated the impact of COVID-19 infection on AITDs that in turn are associated with thyrotoxicosis. A systematic review comprising a total of 13 studies published between December 2019 and October 2021, reported 14 cases of GD and 5 cases of hypothyroidism due to HT occurring concomitantly or following COVID-19 [12]. In particular, eight cases were diagnosed with GD, the most frequent cause of hyperthyroidism in iodine-sufficient areas and especially in middle-aged women, characterized by low TSH and raised serum concentrations of thyroid hormones, presence of TSH receptor antibodies and thyroid stimulating immunoglobulins, and hypoechoic pattern and increased vascularity at ultrasound [12,95]. Four cases also had Graves’ ophthalmopathy (GO), the most common GD extrathyroidal manifestation [96,97,98,99], while more than half of the cases with GD-associated COVID-19 had a previous history of GD or hyperthyroidism [12]. All subjects were treated with antithyroid drugs, mainly methimazole, with most cases showing symptom recovery and thyroid function improvement after therapy [12]. However, despite administering adequate treatment for four patients manifesting thyroid storm (a severe thyrotoxicosis that may cause high mortality even among patients without COVID-19 infection), one patient died of acute respiratory distress syndrome [99,100]. GD is likely a multifactorial disease, and the complex interplay between genetic and non-genetic factors (e.g., iodine, infections, psychological stress, gender, smoking, vitamin D, selenium, immune modulating agents) underlying this condition may cause activation of an immune response involving innate and adaptive immune pathways [50,98]. Notably, the hyperinflammatory status associated with COVID-19 seems to be primarily mediated by T helper (Th) 1-type cytokines as well as IL-6, and a prevalent Th1 immune response (not related to the hyperthyroidism per se, but to the autoimmune process) has also been reported in the immune-pathogenesis of GD [97,101]. On the other hand, the confounder effect of certain drugs should be considered, as reported above. Indeed, while glucocorticoids, heparin and dopamine interfere with HPT function inhibiting the secretion of TSH, NSAIDs administration may result in a transient elevation of thyroid hormones by inducing their displacement from plasma-binding proteins [96,102]. Furthermore, drugs such as tocilizumab which acts as an antibody IL-6 receptor inhibitor, while preventing autoimmune inflammatory responses, might cause some adverse effects that could be confused with the possible onset of thyroid autoimmunity [17,103].

Of the five HT cases included in the systematic review by Tutal et al. (2022) [12], two had previous hypothyroidism. All subjects were treated with LT4 and euthyroidism was achieved within two weeks to four months [12]; however, one patient, diagnosed with myxedema coma, a life-threatening and emergency presentation of hypothyroidism, with concomitant COVID-19, died of cardiac arrest [104,105]. In light of the temporal relationship between COVID-19 infection and onset of HT (concomitant or up to a few weeks; [12]), a possible link between HT and SARS-CoV-2 is based on the hypothesis that the inflammatory status and the consequent oxidative stress from COVID-19 infection determines an impaired signaling in the SIRT1 (a NAD+-dependent Class III deacetylase enzyme) pathway, which in turn results in an altered expression of the transcription factor Forkhead Box P3 (FOXP3) [106,107]. Therefore, as Foxp3 is a critical regulator of the development and function of CD4+CD25+ regulatory T cells (Tregs) and affected Tregs lead to an autoimmune response involving autoreactive T cells, this environment may promote HT development [55,108,109]. In the reassessment of 122 non-critically ill patients after a median interval of 90 days from COVID-19, Lui et al. (2021) [110] observed that anti-TPO titers and anti-TG titers increased significantly and four patients became positive for anti-TPO, evidencing the utility of thyroid function surveillance post COVID-19 and longer follow-up to monitor potential incidents of thyroid dysfunction among COVID-19 survivors.

### 3.4. SARS-CoV-2 Vaccine-Associated Thyroid Disorders

In previous chapters, we attempted to summarize the current evidence on the association between COVID-19 infection and the onset of thyroid dysfunction. However, recent studies have reported emerging findings on SAT occurrence also following SARS-CoV-2 vaccination and some possible mechanisms have been proposed to support this relationship (Table 3) [111]. The first of these is based on the effects produced by adjuvants, immunological or pharmacological substances contained in vaccines, which, if they enhance the response to vaccination, may lead to the insurgence of serious side effects, called “autoimmune/inflammatory syndrome by adjuvants” (ASIA) or Shoenfeld’s syndrome in genetically susceptible and predisposed individuals [112]. ASIA originates from dysregulation of both the innate and adaptive immune systems and is responsible for several autoimmune diseases including systemic lupus erythematosus, rheumatoid arthritis, or autoimmune endocrinopathies like HT and SAT, as described after administration of other vaccines, such as the influenza, human papillomavirus and hepatitis B [112,113]. A second more convincing hypothesis is related to molecular mimicry, as demonstrated by an in vitro study revealing a strong cross-reactivity between antibodies against protein S (the protein that binds to ACE2 receptors and allows the virus to enter the cell) and a number of antigens, including TPO, which could account for autoimmunity and inflammatory reactions observed in both SARS-CoV-2 infection and vaccination in genetically predisposed individuals [111,114,115]. In a systematic review of 30 studies, for a total of 51 patients with diagnosis of SAT having received a recent (within 4 weeks) injection of SARS-CoV-2 vaccine, 74.5% were women with a median age at onset of 40 years, and most subjects were vaccinated with an mRNA vaccine [111]. Another review of the literature (16 reports with 22 SAT cases after vaccination against COVID-19), in addition to confirming the greatest apparent vulnerability of females, reported that the median number of days for the development of symptoms after vaccination was 7 days, and approximately 2 weeks from the onset of symptoms to diagnosis [116]. Therefore, symptoms of thyroiditis appear in a few days, unlike the SAT triggered by COVID-19 infection, which commonly develops within 2–3 weeks from infection, and Classical-SAT that has been recognized to manifest 2–8 weeks after upper respiratory tract viral infections [117,118]. The early presentation of SAT associated with the SARS-CoV-2 vaccination could be a consequence of the maximal concentration of viral proteins, and subsequently of autoimmunity, reached within a few days after vaccination [119,120]. Clinical symptoms recorded were those of painful SAT, namely neck pain, palpitations, fatigue, fever, weight loss, anxiety, or insomnia, accompanied by TSH suppression (88.2% of cases), and increased CRP and ESR, the latter significantly associated with the severity of thyrotoxicosis. Different findings were shown in a retrospective cohort study which included 23 patients with SAT detected within 90 days of a COVID-19 vaccination (CoronaVac or Pfizer/BioNTech) and grouped as Vac-SAT, and 62 patients with SAT detected before the COVID-19 pandemic and grouped as Classical-SAT [121]. The authors found that, among the inflammatory markers, only ESR was significantly higher in the Classical-SAT group than the Vac-SAT group, while the others (e.g., CRP, NLR, platelet to lymphocyte ratio) were similar between the two groups [121]. Moreover, SAT-duration was 28 (10–150) days, and higher in Vac-SAT than in Classical-SAT (*p* = 0.023), while a previous history of LT4 use and increased TSH after resolution were more frequent in Vac-SAT than in Classical-SAT (*p* = 0.027 and *p* = 0.041, respectively). Consistently, Ippolito et al., (2022) [111] reported that hypothyroidism was detected in about 26% of the patients after remission of SAT associated with COVID-19 vaccine, and LT4 was indicated in approximately 60% of them. In addition, same as SAT triggered by COVID-19 infection, patients were treated with NSAIDs, beta blockers and corticosteroids for a median of four weeks, leading to a substantial decrease of patients with thyrotoxicosis at follow-up (31.6%) [111]. Of note, while thyroid function and inflammation did not vary with vaccine type, there was a significant difference in geographic origin, with mostly Asians among the patients receiving non-mRNA vaccines [111]. On the other hand, all patients with a history of autoimmune thyroid disease and who developed SAT, had received the mRNA vaccine [111], which, compared to the other traditional vaccines, is encapsulated by lipid nanoparticles that preserve and maintain mRNA stability and could also have adjuvant capacity [115,122]. Adjuvants have the ability to enhance the half-life and efficacy of therapeutic molecules in cells by increasing the immunogenicity of the active ingredient but, at the same time, some of them (e.g., polyethylene glycol) might induce immune responses in predisposed individuals, including anaphylactic reactions [123,124].

Notably, the SARS-CoV-2 vaccination may precipitate a thyrotoxicosis with different underlying etiologies, as shown by Pla Peris et al. (2022) [118] who reported eight cases with thyrotoxicosis after SARS-CoV-2 vaccination—four cases of GD, two of SAT, one case of concurrent GD and SAT and one of atypical SAT—with the onset of symptoms following vaccination ranging from 10 to 14 days in six out of eight patients and 7–8 weeks in two patients. The other two cases of GD were observed in two female health care workers who had received a SARS-CoV-2 vaccine and three days later manifested clinical signs of thyroid hyperactivity, with increased thyroid hormone levels, suppressed TSH, and elevated antithyroid antibodies [125]. More recently, Chaudhary et al. (2022) [126] published a case series of four patients developing GD following the administration of SARS-CoV-2 vector vaccine. Three cases were females and had a family/self-history of autoimmune disease, and, although all patients responded well to medical treatment and became euthyroid after two to four months, two of them showed worsening of thyrotoxicosis after the second dose of vaccine, demonstrating that further vaccination may result in an amplification of the autoimmune response [126]. Concurrent with hyperthyroidism, two of these patients had mild thyroid eye disease, a debilitating condition that occurs frequently in patients suffering from GD and appears to be more common in individuals of Asian ethnicity [126,127]. In summary, like SAT, GD may also present both after infection with SARS-CoV-2 and vaccine against SARS-CoV-2, suggesting that protein S has the potential to induce or unmask autoimmunity in genetically predisposed individuals, although it is still unclear if its onset may also depend on the bystander effect of adjuvants [126]. Furthermore, to date, cases of GD after SARS-CoV-2 vaccine administration have only been observed with mRNA and viral vector vaccine, but not following inactivated vaccine [127,128].

Although the number of cases of thyroid disorders (the main clinical and biochemical characteristics of which are listed in Table 4), especially SAT, recorded following COVID-19 vaccination is negligible compared to the billions of vaccine doses administered at a global level, their incidence could be underestimated due to a general lack of awareness among clinicians, as well as the possible misdiagnosis of these conditions that could be confused with other side effects occurring after vaccination. Furthermore, while the association between SARS-CoV-2 vaccination and SAT is far from proven and a better understanding of etiopathogenesis will contribute to optimizing patient management, it should be considered that the reported cases have shown a temporal relationship with vaccination, which probably excludes the possibility of other triggering factors. On the other hand, if thyroid disorders following COVID-19 vaccination are a clinical event apparently with a time association with vaccine administration, this does not imply a cause–effect relationship.

## 4. Conclusions. Thyroid and Viral Infection: Any Definitive Evidence of a Causal Relationship or a Casual Association?

**Figure 2:**
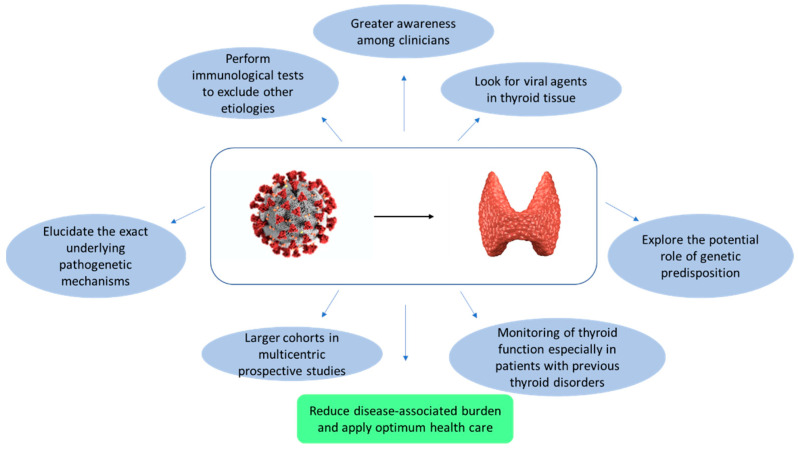
Actions to be taken to verify the nature and strength of the relationship between COVID-19 (or other viral infections) and thyroid disorders.

There are many clues that identify viral infection as the culprit of thyroid dysfunction (more evidence for HCV), although many findings were sporadic and inadequate to support a strong chain of events and connections [67]. In this context, the spread of COVID-19 has provided a substantial increase in evidence that has delved into different aspects of the disease, strengthening the possibility of a relationship between the prevalence of thyroid disorders and infection. Accordingly, a recent meta-analysis showed a high prevalence of thyroid dysfunction in COVID-19 patients, and a risk of abnormal thyroid function tests 3.77-fold higher in patients presenting severe compared to mild to moderate COVID-19 disease [129]. In particular, a number of findings support a close association between COVID-19 disease and SAT, although this complication may be difficult to identify in time due to the possible absence of Classical-SAT symptoms, as well as a crossing of common clinical features between COVID-19 and thyrotoxicosis. However, the amount of data available about SARS-CoV-2 or other viral infections is still not sufficiently exhaustive to establish a definitive proof of the causal relationship between viruses and thyroid dysfunction, and further aspects still need to be elucidated to verify the nature and strength of these associations in order to help plan future preventive and therapeutic strategies for more personal and targeted care with attention to the underlying causes of thyroid dysfunction (Figure 2).

Due to these limitations, it is noteworthy that tests for thyroid function in the setting of COVID-19 are not recommended in the World Health Organization guidelines for COVID-19 clinical management [130]. Nonetheless, given the laboratory availability of these tests, their low cost and relevance to potential co-occurrence of thyrotoxicosis caused by destructive thyroiditis related to SARS-CoV-2 and/or NTIS, routine assessment of thyroid function in COVID-19 patients (especially those admitted to HICUs), should be considered and could also be a reasonable strategy in case of other viral infections.

## Figures and Tables

**Table 1:** Definition of the major thyroid disorders associated to viral infections.

Thyroid Disorder	Definition	Reference
Subacute thyroiditis	Inflammatory disorder of the thyroid gland with characteristic presentation and clinical course; it can be classic (painful) or atypical (painless).	[2]
Nonthyroidal illness syndrome	Condition of the thyroid associated to changes in serum thyroid hormone levels observed in critically ill patients in the absence of hypothalamic–pituitary–thyroid primary dysfunction.	[3]
Autoimmune thyroid diseases	The most prevalent organ-specific autoimmune diseases due to lymphocytic infiltration that causes tissue damage and alters the function of the thyroid gland.	[4]
Graves’s disease	Autoimmune disorder derived from stimulation of the TSH receptor located on the thyroid gland by TSH receptor antibody, which may result in hyperplasia and hyperfunction of the thyroid gland.	[5]
Hashimoto disease	Autoimmune disorder caused by the presence of TG and TPO autoantibodies leading to thyrocyte destruction, including apoptosis.	[5]

Abbreviations: TG: thyroglobulin; TPO: thyroid peroxidase; TSH: thyroid stimulating hormone.

**Table 2:** Main elements that support and weaken the relationship between viral infection and thyroid dysfunction/disease.

Evidence for the Relationship	References	Lack of Evidence
Presence of different viruses in thyroid tissue.	[29]	The virus can remain in the tissue without any evidence of systemic presence.
Both ACE2 and TMPRSS2 mRNAs and proteins detected in the thyroid tissue.	[16,17]	Most available data based on serological measurements; lack of information on the presence and copy numbers of viral genome.
T3 and T4 modulators of immune responses (both innate and adaptive) and regulators of chemokine gene expression.	[22,23]	Presence of antibodies do not directly reflect viral status (high levels may persist long after viral eradication, without correlation to disease severity or duration).
Seasonal pattern of SAT in parallel with outbreaks of viral infection (e.g., enterovirus).	[29,30]	The presence of antibodies against a virus does not mean that this infection necessarily causes disease, especially if the virus is common in the population.
Secretion of pro-inflammatory cytokines and chemokines, immune deficiency related to infection, destruction of lymphocytes, inhibition of the innate immune response and direct destruction of follicular cells with apoptosis.	[35]	Specific antibodies do not necessarily imply active infection, as positivity for antibodies may remain after viral clearance.
Association (e.g., SAT) with haplotypes, as markers of genetic susceptibility.	[34,36]	Thyroid dysfunction not developed in all infected subjects: triggering or worsening autoimmunity in genetically predisposed individuals, thus only/more in susceptible subjects.
Molecular mimicry (the virus may mimic the structure of some components of the thyroid, initiating autoimmune responses).	[62]	Lack of demonstration of the relationship between clinical status (e.g., same prevalence of the viral pathogens between AITDs patients and controls), and data on the role of coinfection or the presence of susceptibility genes.
Enhanced processing and presentation of auto-antigens by antigen-presenting cells (e.g., changes in self antigen expression).	[62]	
Increased inflammation and cytokine release (activation of autoreactive T cells).	[62]	
Activation of lymphocytes by lymphotropic viruses (e.g., increased generation of circulating immune complexes).	[62]	

Abbreviations: ACE2: angiotensin-converting enzyme 2; AITDs: autoimmune thyroid diseases; SAT: subacute thyroiditis; T3: 3,3′,5-triiodo-L-thyronine; T4: L-thyroxine; TMPRSS2: transmembrane serine protease 2.

**Table 3:** Summary of findings on the relationship between SARS-CoV-2 vaccination and thyroid disorders.

Study Design	Population	Type of Vaccine	Thyroid Disorder	Main Results	Reference
Systematic review of case report and case series + 3 original cases	51 cases (78.5% women), median age 39.5 years. 47% European, 35.3% Asian, 15.7% North American, 2% Australian	66% mRNA vaccine; 18% viral vector vaccine; 12% inactivated virus; 4% heterologous vaccination	SAT	Onset of SAT after a median of 10 days (range 4–14 days) after the vaccine shot. Thyrotoxicosis in 88.2% of patients, decreasing at 31.6% at a 4–8-week follow-up. 26.3% of patient hypothyroid at follow-up. Corticosteroids used in 56.4% of treated patients; LT4 used in 60% of hypothyroid patients. Patients undergoing non-mRNA vaccines most frequently Asian.	[111]
Case report and literature review: (16 reports)	Case report: 34-year-old male Literature review: 23 cases (18 females), median age 40 years	Case report: Moderna Literature review: AstraZeneca (6 cases); CoronaVac (5 cases); Pfizer-BioNTech (4 cases); Moderna (3 cases); Janssen (1 case); Covaxin (1 case); no vaccine reported (3 cases)	SAT	Case report: First symptoms 5 days after the first dose of the vaccine. Diagnosis of thyrotoxicosis. Treatment with steroid therapy (in the meantime second dose of vaccine). Literature review: Median 7 days (range 5–21 days) for the development of symptoms after vaccination. Median 2 weeks (range 6 days–10 weeks) from the onset of symptoms to the diagnosis. 13 out of 23 patients requiring steroid therapy.	[116]
Single-center case series	8 cases (5 females). Mean age 56.6 years. 7 patients with normal thyroid function before vaccination. None with family history of thyroid disease. 1 patient with subclinical hypothyroidism, with no data of thyroid antibodies	Pfizer BNT162b2 mRNA vaccine (5 cases) Moderna 1273 mRNA vaccine (3 cases)	GD (4 cases) SAT (2 cases) GD + SAT (1 case) Atypical SAT (1 case)	Onset of symptoms following vaccination ranging from 10 to 14 days in 6 patients and 7–8 weeks in 2 patients. None of the patients with GD had Graves’ orbitopathy. All patients with thyrotoxicosis after vaccination. Patients with SAT and atypical SAT treated with NSAIDs. Patients with GD and those with concurrent GD and SAT treated with methimazole and NSAIDs, respectively. All patients were still being followed up when the study was published.	[118]
Observational, retrospective cohort study	23 patients in the Vac-SAT group (18 in BioNTech- SAT and 5 in CoronaVac-SAT) and 62 patients in the Classical-SAT group. Median age 42 and 43 years, respectively. Female/male ratio 63.5/36.5% (54/31), and similar between the groups	CoronaVac, BioNTech- SAT	Vac-SAT (SAT diagnosed in the first 90 days after COVID-19 vaccination) Classical-SAT (detected after upper respiratory tract infection before the COVID-19 pandemic)	Median SAT-duration was 28 (10–150) days in total; higher in the Vac-SAT group than in the Classical-SAT group (*p* = 0.023). SAT developed after first dose of vaccine in 5 patients in Vac-SAT, 2 patients in CoronaVac-SAT, and 3 patients in BioNTech-SAT groups (*p* = 0.263). T2D present only in the BioNTech-SAT group (*p* < 0.001). LT4 history more frequent in Vac-SAT than in Classical-SAT (*p* = 0.027) but present at a similar frequency in CoronaVac-SAT and BioNTech- SAT groups (*p* = 0.602). TSH elevation more frequent in the Vac-SAT group (*p* = 0.041). All patients treated with anti-inflammatory agents such as NSAIDs alone or methylprednisolone.	[121]
Case report	Case 1: 40-year-old health care worker with arterial hypertension and a history of COVID-19 Case 2: 28-year-old medical resident with no history of autoimmune endocrine diseases	Pfizer-BioNTech	GD	Case 1 Symptoms 2 days after vaccination. Episodes of parozysmal atrial fibrillation. Treatment with proponalol, diltiazem, ivabradine and thiamazole. Case 2. Symptoms 3 days after vaccination. Treatment with proponalol and thiamazole. No signs of dermopathy, orbitopathy or other undifferentiated connective disease in either patient.	[125]
Single-center case series	Case 1: 20-year-old male, without previous history of thyroid disease Case 2: 46-year-old female, without previous history of thyroid disease (history of autoimmune hypothyroidism in the patient’s son) Case 3: 19-year-old female, with a family history of autoimmune hypothyroidism in the mother and GD in the father Case 4: 37-year-old female with a history of autoimmune hypothyroidism (history of autoimmune hypothyroidism in the mother)	Viral vector vaccine ChAdox1nCoV-19	GD	All patients with thyrotoxicosis treated with carbimazole and propranolol. Cases 1 and 2: diagnosis of TED. Cases 1,3: no further worsening of TFT or TED after a second dose of vaccine. Case 2: after a second dose of the vaccine, reappearance of symptoms of thyrotoxicosis along with worsening of TFT but not of TED. Case 4: normalization of TFT but no second dose of vaccine.	[126]

LT4: levothyroxine; NSAID: nonsteroidal anti-inflammatory drug; SAT: subacute thyroiditis; T2D: type 2 diabetes; TED: thyroid eye disease; TFT: thyroid function test; TSH: thyroid stimulating hormone.

**Table 4:** Summary of main clinical and biohumoral findings in thyroid disorders after SARS-CoV-2 infection and vaccination.

Thyroid Disorder	Clinical Signs	Biohumoral Features	References
SAT	Thyrotoxicosis: fever, anterior neck pain, fatigue, tremors, anosmia, sweating, palpitations, weight loss, anxiety, insomnia. In COVID-19 patients, possibility of absence of neck pain (atypical SAT).	During thyrotoxicosis, high free T4 levels and low to undetectable TSH levels. Also normal TSH levels possible. In subjects with overt thyrotoxicosis, higher serum free T4 levels than those with subclinical disorder. In a minority of subjects, including those admitted to intensive care units, absence of thyrotoxicosis. High ESR, high CRP (associated with the severity of thyrotoxicosis), increase in white blood count, negativity for thyroid autoantibodies, enlarged hypoechoic thyroid with decreased vascularity found on ultrasound, and markedly reduced or absent iodine uptake in the gland. In painless SAT, lymphopenia instead of lymphocytosis.	[9,11,27,28,73,74,75,78,79,111,116,118,126]
NTIS	Observed in most critically ill patients, being considered a strong predictor of poor prognosis. Also experienced by patients with mild to moderate COVID-19 not requiring intensive care.	Initial reduction in plasma T3 and increased plasma reverse T3 levels without a concomitant rise in TSH. Conditions of persistent illnesses led to a global reduction of TSH levels, free T3 and free T4. The degree of decrease in TSH and total T3 levels positively correlated with disease severity. Lower free T3/free T4 ratio, serum albumin and lymphocyte count, increased absolute neutrophil count and NLR, higher inflammatory markers (CRP, ESR and procalcitonin), higher indices of tissue injury (i.e., aspartate aminotransferase and lactate dehydrogenase, ferritin, high-sensitivity cardiac troponin I), increased levels of coagulation serum markers (prothrombin time, fibrinogen, D-dimer), higher radiological scores of disease severity (Lung Immune Prognostic Index, Sequential Organ Failure Assessment Score and Tomographic severity score). Significant inverse correlation observed between fT3 serum levels and hydration status.	[16,40,41,42,43,85,86,87,88,89,90,91,92,93]
AITDs	In GD, enlargement of the thyroid (goiter) Possibility of co-occurrence of Graves’ ophthalmopathy in COVID-29 patients and mild thyroid eye disease following SARS-CoV-2 vaccination.	In GD, low TSH and raised serum concentrations of thyroid hormones, presence of TSH receptor antibodies and thyroid stimulating immunoglobulins. Possibility of increase in anti-TPO and anti-TG antibody levels. Hypoechoic pattern and increased vascularity at ultrasound. In HT, infiltration of autoreactive T cells in thyroid tissues and production of autoantibodies (anti-TPO and anti-TG) associated with destruction of the thyroid follicles, which ultimately results in hypothyroidism (i.e., low serum free T4 and elevated serum TSH levels).	[12,55,56,95,96,97,98,99,118,125,126,127]

Abbreviations: AITDs: autoimmune thyroid diseases; CRP: C-reactive protein; ESR: erythrocyte sedimentation rate; fT3: free 3,3′,5-triiodo-L-thyronine; GD: Graves’ disease; HT: Hashimoto’s thyroiditis; NLR: neutrophil to lymphocyte ratio; NTIS: nonthyroidal illness; SAT: subacute thyroiditis; T3: 3,3′,5-triiodo-L-thyronine; T4: L-thyroxine; TG: thyroglobulin; TPO: thyroid peroxidase; TSH: thyroid stimulating hormone.

## Data Availability

Not applicable.

## Abbreviations

ACE2	Angiotensin-converting enzyme 2
AITDs	Autoimmune thyroid diseases
CRP	C-reactive protein
ESR	Erythrocyte sedimentation rate
fT3	Free 3,3′,5-triiodo-thyronine
GD	Grave’s disease
HT	Hashimoto’s thyroiditis
IL-6	Interleukin-6
LT4	Levothyroxine
NLR	Neutrophil-lymphocyte ratio
NSAIDs	Nonsteroidal anti-inflammatory drugs
NTIS	Nonthyroidal illness syndrome
SAT	Subacute thyroiditis
T3	3,3′,5-triiodo-thyronine
T4	L-thyroxine
TG	Thyroglobulin
TNF-α	Tumor necrosis factor alpha
TPO	Thyroid peroxidase
TSH	Thyroid stimulating hormone
